# Interactive Narrative Simulation as a Method for Preceptor Development

**DOI:** 10.3390/pharmacy10010005

**Published:** 2021-12-28

**Authors:** Charlene R. Williams, Robert Hubal, Michael D. Wolcott, Abbey Kruse

**Affiliations:** 1 The Eshelman School of Pharmacy, The University of North Carolina Chapel Hill, Asheville, NC 28804, USA; 2 The Eshelman School of Pharmacy, The University of North Carolina Chapel Hill, Chapel Hill, NC 27599, USA; hubal@unc.edu (R.H.); wolcottm@email.unc.edu (M.D.W.); abbeykruse@unc.edu (A.K.)

**Keywords:** preceptor development, simulation, situational judgment tests

## Abstract

(1) Background: This proof-of-concept study assessed an interactive web-based tool simulating three challenging non-academic learning situations—student professionalism, cross-cultural interactions, and student well-being—as a means of preceptor development. (2) Methods: Three scripts focused on professionalism, cross-cultural interactions, and student well-being were developed and implemented using a commercial narrative tool with branching dialog. Delivered online, this tool presented each challenge to participants. Participants had up to four response options at each turn of the conversation; the choice of response influenced the subsequent conversation, including coaching provided at the resolution of the situation. Participants were invited to complete pre-activity, immediate post-activity, and one-month follow-up questionnaires to assess satisfaction, self-efficacy, engagement, and knowledge change with the tool. Knowledge was assessed through situational judgment tests (SJTs). (3) Results: Thirty-two pharmacist preceptors participated. The frequency of participants reflecting on challenging learning situations increased significantly one-month post-simulation. Participants affirmatively responded that the tool was time-efficient, represented similar challenges they encountered in precepting, was easily navigable, and resulted in learning. Self-efficacy with skills in managing challenging learning situations increased significantly immediately post-simulation and at a one-month follow-up. Knowledge as measured through SJTs was not significantly changed. (4) Conclusions: Preceptors found an interactive narrative simulation a relevant, time-efficient approach for preceptor development for challenging non-academic learning situations. Post-simulation, preceptors more frequently reflected on challenging learning situations, implying behavior change. Self-efficacy and self-report of knowledge increased. Future research is needed regarding knowledge assessments.

## 1. Introduction

Health professions educators continually seek new and effective methods to engage preceptors. Preceptors, when surveyed, show greater preference for online versus live training due to flexibility, while self-study is valued due to accessibility [1,2]. Consistency in presentation is important to ensure a base level of competency, yet there are benefits to providing training in varied formats since not all preceptors respond similarly to the same media [2,3]. However, more data on effective methods for preceptor development are needed, as a preferred delivery method based on learning outcomes has not yet been identified [2,4,5,6,7].

Simulation through virtual role-playing represents an attractive research area for preceptor development due to its accessibility, interactivity, adaptability, and availability in self-study formats [8]. Simulated environments have been used extensively within health professions to allow students to practice professionalism skills with simulated patients [9,10,11,12]. Though type of technology varies considerably among these studies, findings generally indicate that students engage in interactions with simulated patients and apply learned skills more broadly [13,14,15,16]. Simulation may also help students attain and retain educational goals due to its active nature and self-directed format [17].

Virtual or screen-based simulation has also been studied for other uses—such as for social interaction, change management, stress management, and job interview skills—due to its abilities to offer repetitive practice, individualization, immediate feedback, and an interpersonal safe environment [18,19,20,21,22]. It also addresses some limitations of accessibility and resource requirements with in-person simulation [21,22]. Furthermore, serious games are a type of simulation-based education to develop nontechnical abilities such as decision-making skills [23].

Screen-based simulation also allows for recreating situations that may be difficult or infeasible in person due to discomfort with participating in role play, inability to attend live training, or availability of facilitators. This capability could potentially be used to train preceptors for challenging non-academic learning situations such as professionalism concerns. Given preceptors’ level of involvement in pharmacy curricula, it is expected that preceptors may frequently encounter such situations [24,25,26]. Actor-based in-person role-plays have been used effectively as a type of simulation for nurse preceptor development in difficult situations [27]. The scenarios were included as part of a preceptor training program for new preceptors that also had a didactic component. The scenarios included developing a precepting plan, implementing the plan, and delivering feedback. Half of the participants had the opportunity to play the role of the preceptor while the rest of the participants observed. Learning outcomes were assessed with pre-post knowledge questions and were significantly improved after the program, and preceptors positively received the program. A limitation was the time commitment of faculty. Since the training included didactic content in addition to the simulation, it is unknown what contribution simulation alone made to knowledge change [27]. A web-based simulation may increase preceptors’ accessibility to training that includes application with real-world scenarios and would not require faculty oversight. While computer-based simulation has been used as a viable training modality in health education curricula [28,29], it is unknown if this translates to preceptors.

This proof-of-concept study evaluated narrative-based simulation using a web-based tool designed to prepare preceptors for managing challenging non-academic learning situations. The simulation included three situations that preceptors may encounter in practice: professionalism, cross-cultural interactions, and student well-being. The research aims were to determine if simulation is an effective method for increasing preceptor knowledge, behavior, and self-efficacy, and to gauge level of preceptor engagement and satisfaction with simulation.

## 2. Materials and Methods

Three challenging situations were created based on topics frequently offered in preceptor development programs, situations reported to the authors’ School, and topic requests from School preceptors [2]. Challenging non-academic learning situations were defined as situations demanding preceptor intervention; intervention is prompted due to concern about the student’s performance or well-being and requires applying skills to facilitate interaction beyond the traditional role of preceptor as educator only [25]. The situations involved professionalism (tardiness), cross-cultural interactions (patient refusing care), and student well-being (noticeable changes in emotional state). Literature on challenging learning situations, remediation, feedback, well-being, and cross-cultural interactions informed script development, coaching, and creation of supplemental (reference) resources [25,26,30,31,32,33,34,35,36,37,38,39,40,41,42,43,44].

Scripts were implemented using the ink editor from Inkle Studios (Cambridge, UK). This narrative tool enables rapid writing and testing of branching dialog. In prior studies [45,46], different organization methods of dialog were used, including a dynamic main menu and simple linear flow. The structure used here was mixed: dialog had set flow from start (introduction of the situation with the student) to finish (resolution of the situation); in between, at each turn in the conversation, participants could choose from up to four options of how to address or respond to the student. Response options were created by three subject matter experts. The presentation was primarily text-based and required menu selections to progress. The program provided textual feedback at resolution of each challenge based on participant choices. This individualized coaching summarized what participants did well and what could be improved on instead of indicating whether their response was correct or incorrect. The simulation allowed participants to explore the merits and challenges to potential solutions. General written strategies from the literature and topic experts were provided to all participants regardless of simulation performance.

A convenience sample of participants was obtained by sending an email request for voluntary participation to 1275 preceptors at one school of pharmacy. Each participant had an individual session with the tool and research team member, either in person or via Zoom (San Jose, CA, USA). Sessions were audio-recorded. Participants were asked to think aloud as they read descriptions and selected among options, providing a steady flow of detail of their observations and reasoning [47]. The three situations could be completed in any order, and participants could replay or review challenges as often as they liked. Sessions were untimed, generally requiring 45 min to one hour total.

Three Kirkpatrick levels of training evaluation were considered in the outcome assessments: reaction, learning, and behavior [48]. All participants were asked to complete three questionnaires: pre-activity (prior to running through challenges), post-activity (immediately after), and one-month follow-up. All questionnaires were accessed online using Qualtrics (Provo, UT, USA). The pre-activity questionnaire included demographics, the frequency participants encountered challenging learning situations, their perceived behaviors, self-efficacy, and knowledge of managing these situations. Situational judgment tests (SJTs) informed design of knowledge questions, presenting three additional situations that participants may encounter. For the eight-item knowledge test, participants were instructed to prioritize potential responses in order of most to least favored [49].

The immediate post-activity questionnaire asked participants to rate satisfaction with the simulation, then through open-ended questions sought features liked most and least about the tool, suggestions for improvement, and desired additional resources. Participants were given the same self-efficacy, perceived behaviors, and knowledge questions. The one-month follow-up questionnaire included questions on perceived precepting behavior with challenging learning situations, whether coaching from the simulation had been used, and resources still needed. Participants also rated their self-efficacy, described behaviors, and completed the knowledge questions. The self-efficacy instrument was designed, using a five-point scale anchored by ‘strongly disagree’ and ‘strongly agree’, in accordance with recognized design principles and reviewed by education research and pharmacy practice experts before administration [50]. Descriptive statistics were utilized to assess demographics. For knowledge questions, participants were asked to rank solutions for the presented scenarios from a list of four responses from most to least favored; correct answers were awarded one point and based on a key from subject-matter experts (three investigators with precepting and education expertise and eight preceptor faculty with five-plus years’ precepting experience). Paired comparisons were performed using *t*-tests when a sufficient sample size and normality of distribution was warranted, otherwise Wilcoxon signed rank tests were utilized, with significance set at 0.05, entries removed that had missing values, and Bonferroni correction for multiple tests. Qualitative data were analyzed using informal methods with patterns sought among participant responses to open-ended questions. The study was reviewed and deemed exempt by the School’s Institutional Review Board.

## 3. Results

Thirty-two preceptors were recruited to test the simulation tool (Table 1). Of these participants, 75% (N = 24) and 78% (N = 25) completed the immediate and one-month follow-up questionnaires, respectively.

Percentages of participants responding that they addressed tardiness, cross-cultural interactions, or mental health with students at least monthly prior to the simulation were 53% (N = 17), 50% (N = 16), and 38% (N = 12), respectively. Over their careers, 66% (N = 21), 53% (N = 17), and 53% (N = 17) indicated they had addressed these concerns. On a question asking about preceptor behavior related to regularly reflecting on challenging situations of all kinds with learners, 66% (N = 21) answered in the affirmative (agreeing or strongly agreeing) prior to the simulation experience while 80% (N = 20) answered so one-month after participation; a signed rank test comparing paired pre-activity and post-activity responses indicated that post-activity responses were significantly higher than pre-activity responses (*p* < 0.04). Participants’ beliefs in their ability to successfully address challenging situations also changed, using the same test, from pre-activity to one-month post (75%, N = 24 vs. 80%, N = 20, *p* < 0.05). Forty-two percent (N = 10) in the one-month post-activity survey indicated they had used strategies from the tool.

Regarding usability, 92% (N = 22) of participants answered positively about time-efficiency and 92% (N = 22) felt navigation was easy. All agreed or strongly agreed that the experience was applicable to their practice, and 79% (N = 19) noted the challenges were similar to situations they experienced. Nearly all (96%, N = 23) claimed to have learned from using the tool. Open-ended positive comments included realism of challenges, ability to replay and explore dialog progression based on different response options, reflection on reactions, coaching provided at each challenge’s resolution, and engagement and ease of use. Constructive comments included need for a back button (as opposed to replay of the whole challenge), desire to pinpoint which response selection(s) drove feedback (since coaching was provided only at the end of a dialog and may have referred to one or several choices made by the participant), and some vagueness in response options. Forty-two percent (N = 10) of participants disagreed or strongly disagreed that there were right answers to the scenarios in the simulation, debating if just one answer fit, that response options were vague, or how certain answers were not considered “ideal”. Several participants requested additional challenging situations focused on good communication skills and social cues. One participant suggested using the tool to create virtual “rounds” for preceptors to share interactions within the situations and learn from each other how to manage the student. Additional resources requested included discussion with a School mentor, discussion with colleagues, continuing education programs on challenging situations, and handouts outlining resources.

Moreover, participant self-efficacy significantly increased from pre-activity to immediate post-activity (Table 2) and maintained from immediate to one-month later. Results from the SJT-structured knowledge test administered before, immediately after, and at a one-month follow-up suggested no change in participant knowledge (*p* > 0.12; see Table 3). There were no systematic differences in how participants ranked response options across the three questionnaires, reflecting that participants differed among themselves and with experts regarding “right” answers provided to the challenges.

## 4. Discussion

This is one of the first studies to assess the effectiveness of web-based narrative simulation for preceptor development. Its strengths are in measuring preceptor satisfaction, self-reported behaviors, self-efficacy, and knowledge pre-post simulation. The simulation had a positive effect on preceptor self-efficacy and self-perception of knowledge but not on knowledge as measured by SJTs. Overall, most participants found the tool useful and the scenarios realistic; issues seemed to surround the sometimes-vague response options.

Preceptors’ responses to their frequency of addressing challenging non-academic learning situations (particularly tardiness, cross-cultural issues, and mental health concerns) indicated challenges selected for the simulation were relevant. Preceptors agreed these challenges were similar to situations they had experienced and applicable to their practice, indicating a higher prevalence of students requiring intervention than has been cited in the literature [24,25,26]. A potential explanation to this finding is preceptors’ possible historical underreporting [25]. Challenging learning situations and feedback are frequently offered or recommended as topics of preceptor development, and this pilot study validates their inclusion in simulation training [2,51,52].

One-month after simulation, preceptors more frequently reflected on challenging learning situations, implying behavior change. Preceptors’ belief in their ability to successfully manage challenging learning situations increased after the simulation. Self-efficacy measures increased for challenging situations with learners having general professionalism, cultural awareness, and mental health concerns. Preceptors also believed the tool was time-efficient and easily navigable, an important consideration given time constraints that preceptors face [52,53].

Literature is presently lacking in assessment of preceptor self-efficacy with web-based simulation. Satisfaction data are also relatively lacking. A comparison of three simulation modalities (paper, actor, and computer-based) in Master of Pharmacy students found similar satisfaction with all modalities [29]. Though that study was with a student population, similarities in positive aspects of computer-based simulation with the present study include ability to replay or repeat situations, high engagement, and encouragement of reflection. No overlap was found in negative feedback, which could be related to differences in design or in needs between students and preceptors.

Most preceptors reported learning using the web-based simulation tool. However, learning was not demonstrated in any systematic changes to their response prioritizations compared to expert response prioritizations. Participants and experts did not always agree on correct responses to the simulation scenarios nor the knowledge situations in the SJTs. Problem solution may be idiosyncratic; preceptors may learn strategies how to address a challenge, but each individual does so in their own way based on situational constraints. While the dynamic simulation allowed for variability in responses to complex situations, SJTs with a pre-selected best response may not have and therefore may not be optimal for assessing knowledge change for these types of situations. It is also possible that these preceptors were a self-selected group already knowledgeable in managing challenging situations, or that response options were too unclear in how they should be prioritized. Further investigation of the knowledge testing is warranted. There is evidence of learning using role play simulation for nurse educator development when comparing open-ended knowledge questions related to course objectives pre- and post-simulation [27]. Participants in that study believed role play simulation was more effective than lecturing only [27]. Other literature describing knowledge change using simulation for preceptors is not presently available; additional study of best practices with knowledge change assessments in simulation is needed.

This pilot study has several limitations. First, it was conducted with a small number of preceptors at one school of pharmacy to assess proof-of-concept; therefore, caution should be used when applying to other populations of preceptors who may have different experiences. Second, preceptors agreeing to participate may have opted in because they were open to using computer-based technology for preceptor development, perhaps causing bias in favor of simulation. Third, knowledge change assessed through SJTs did not show systematic effect. This form of assessment, in addition, was static, in contrast to the dynamic simulation. In addition, it is unclear if preceptors’ access to other training and resources after simulation could have impacted their self-efficacy. Further study is needed to confirm findings in larger populations of preceptors and determine best practices around use of simulation for training of preceptors in dealing with challenging situations and testing for knowledge change.

## 5. Conclusions

Preceptors responded favorably to an interactive narrative simulation and demonstrated sustained increased self-efficacy with skills related to managing challenging non-academic learning situations from before the simulation to after. Preceptors reported an increase in reflecting on challenging situations. A test of change of knowledge from before to after the simulation did not show systematic effects when comparing participants’ prioritized response options to a SJT to a separate group of experts’ prioritizations. Future work with additional situations, preceptors, and formal knowledge assessments is warranted.

## Figures and Tables

**Table 1:** Demographics of preceptors participating in a simulation for challenging student learning situations.

Characteristic	Participants (N = 32)
**Age**	**Data**
26–35	44% (N = 14)
36–45	31% (N = 10)
46–55	19% (N = 6)
No response	6% (N = 2)
**Degree/Credentials** ^1^	
Bachelor’s degree (e.g., BSPharm, BS, BA)	53% (N = 17)
Master’s degree (e.g., MS, MEd)	16% (N = 5)
Professional degree (e.g., PharmD, MD, JD)	88% (N = 28)
Doctoral degree (e.g., PhD, EdD)	3% (N = 1)
Post-graduate year 1 (PGY1) residency	38% (N = 12)
Post-graduate year 2 (PGY2) residency	38% (N = 12)
Board certification (e.g., BCPS)	53% (N = 17)
**Years Precepting Experience**	
<1 year	9% (N = 3)
1–5 years	53% (N = 17)
6–10 years	6% (N = 2)
11–15 years	6% (N = 2)
16–20 years	13% (N = 4)
>20 years	13% (N = 4)
**Number of Students Precepted Per Year**	
1–5	59% (N = 19)
6–10	13% (N = 4)
11–15	6% (N = 2)
No response	22% (N = 7)
**Number of Residents Precepted Per Year**	
0	47% (N = 15)
1–5	41% (N = 13)
6–10	3% (N = 1)
11–15	3% (N = 1)
No response	6% (N = 2)
**Type of Student Precepted** ^1^	
First-year student pharmacists	34% (N = 11)
Second-year student pharmacists	50% (N = 16)
Third-year student pharmacists	59% (N = 19)
Fourth-year student pharmacists	94% (N = 30)
First-year pharmacy residents	50% (N = 16)
Second-year pharmacy residents	41% (N = 13)
Other health professions students (physician, physician assistant, etc.)	19% (N = 6)
**Previous Preceptor Training and Continuing Education Utilized** ^1^	
School-based preceptor training and development programming	91% (N = 29)
Preceptor development programs for resident preceptors at the organization	56% (N = 18)
Attendance at national pharmacy preceptor’s conference	9% (N = 3)
Continuing education seminars/webinars/workshops	78% (N = 25)
Pharmacist letter resources	31% (N = 10)
Professional organization resources	34% (N = 11)
Preceptor development books	6% (N = 2)
Other national training program	6% (N = 2)

^1^ Respondents could select all that apply.

**Table 2:** Preceptor degree of confidence (self-efficacy) responding to challenging situations.

	Confidence (i.e., “____ Percent of the Time I Am Confident I Can...”) (0—Not Confident; 100—Highly Confident)	Pre-Activity Mean (N = 32) (SD)	Immediate Post Mean (N = 24) (SD)	One-Month Follow-Up Mean (N = 25) (SD)
General activities	Recognize challenging learning situations	79.4 (13.7)	85.0 (12.5)	87.6 (8.8)
	Discuss challenging situations with students	70.0 (15.9)	80.0 (16.7)	81.2 (10.5)
	Understand strategies to address challenging situations with students	62.9 (15.1)	73.3 (15.8)	79.2 (10.4)
	Identify resources to help address challenging situations with students	55.0 (21.1)	73.3 (18.1)	72.8 (15.9)
	Reflect on opportunities to improve how I address challenging situations with students	68.8 (21.8)	82.5 (16.2)	86.0 (12.2)
General activities average		67.2 (19.4)	78.8 (16.4) ^1^	81.4 (12.8) ^2,4^
Professionalism	Identify issues with professionalism among students	86.3 (12.4)	90.0 (9.3)	93.6 (7.0)
	Discuss professionalism issues with students	80.0 (15.9)	85.4 (13.2)	89.2 (10.8)
	Understand strategies to address professionalism issues with students	71.6 (17.6)	82.5 (13.9)	87.2 (13.1)
	Identify resources to help address professionalism issues with students	59.7 (21.8)	77.9 (20.0)	83.6 (16.6)
	Reflect on opportunities to improve how I address challenging professionalism situations with students	76.3 (16.2)	87.9 (13.5)	89.6 (12.1)
Professionalism average		74.8 (19.1)	84.8 (14.8) ^1^	88.6 (12.5) ^2,3^
Cross-cultural interactions	Define cultural awareness	71.3 (16.0)	79.6 (15.2)	83.6 (13.2)
	Discuss cross-cultural issues with students	63.8 (20.4)	69.6 (19.7)	76.0 (20.0)
	Understand strategies to address cross-cultural issues with students	51.9 (22.9)	68.8 (20.7)	74.0 (18.9)
	Identify resources to help address cross-cultural issues with students	39.7 (21.5)	68.3 (24.3)	68.0 (21.4)
	Reflect on opportunities to improve how I address challenging cross-cultural situations with students	60.9 (26.9)	80.0 (19.6)	80.8 (15.8)
Cross-cultural interactions average		57.5 (24.2)	72.3 (20.8) ^1^	76.5 (18.6) ^2,3^
Mental health concerns	Identify mental health concerns with students	67.5 (21.1)	78.8 (18.7)	79.6 (17.2)
	Discuss mental health concerns with students	57.8 (25.5)	69.6 (24.0)	73.2 (22.5)
	Understand strategies to address mental health concerns with students	58.8 (27.0)	70.4 (22.7)	73.2 (21.9)
	Identify resources to help address mental health concerns with students	53.4 (27.1)	71.3 (23.6)	76.8 (20.1)
	Reflect on opportunities to improve how I address mental health concerns with students	65.9 (23.9)	79.6 (24.0)	83.2 (19.3)
Mental health concerns average		60.7 (25.3)	73.9 (22.8) ^1^	77.2 (20.3) ^2,3^

^1^ Difference between immediate post and pre-activity: all *p* < 0.02. ^2^ Difference between follow-up and pre-Activity: all *p* < 0.01. ^3^ Difference between follow-up and immediate post: all *p* < 0.01. ^4^ No difference between follow-up and immediate post; all *p* > 0.05.

**Table 3:** Percent of respondents matching their first prioritized response to SJT questions compared to expert rankings.

	**Scenario 1**			**Scenario 2**		**Scenario 3**		
	Question			Question		Question		
Administration	1	2	3	1	2	1	2	3
Pre-activity	40	53	77	93	90	63	93	50
Immediate post	58	53	74	90	79	53	84	74
One-month follow-up	64	64	68	91	82	73	91	77

## Data Availability

Not applicable.
